# Behavioral Avoidance Response of *Daphnia* to Fungal Infection Caused by *Metschnikowia* Species in a Temperate Reservoir

**DOI:** 10.3390/biology11101409

**Published:** 2022-09-27

**Authors:** Seong-Ki Kim, Jong-Yun Choi

**Affiliations:** National Institute of Ecology, Seo-Cheon Gun 33657, Chungcheongnam-do, Korea

**Keywords:** migration pattern, biological interaction, *Daphnia pulicaria*, defense strategy, dormant eggs, microcosm experiment

## Abstract

**Simple Summary:**

The negative effects of fungal infection on the survival and reproduction of cladocerans, a representative prey community in freshwater, are unexplored. In this study, we hypothesized that the winter migration pattern of *Daphnia pulicaria*, observed in the Anri Reservoir in South Korea, is the host’s defense response to fungal infection. *Daphnia pulicaria* was mainly distributed in the central bottom layer of the reservoir before winter (summer to autumn) but migrated to the littoral area during winter as fungal infections in the communities gradually spread in the bottom region. However, when the spread of infection was low, *D. pulicaria* did not migrate. The migrated individuals with dormant eggs were mostly infected and are believed to have migrated to the littoral area to freeze their dormant eggs. We found that dormant eggs of *D. pulicaria* obtained from ice crystals had lower hatching and infection rates than dormant eggs obtained adaptively in Daphnia mothers. Such a strategy is an efficient response of *D. pulicaria* to avoid the spread of fungal infection in communities and to maintain their continuous population growth.

**Abstract:**

Morphological or behavioral defense mechanisms are important evolutionary strategies for the survival of prey. Studies have focused on predation and competition, but infection has been overlooked, despite being a determining factor of distribution and species diversity of prey. We hypothesized that the winter migration of *Daphnia pulicaria* is a community defense strategy to avoid fungal infection. To test this hypothesis, environmental variables and the Cladocera community, including *D. pulicaria*, were monitored in three study sections of the Anri Reservoir in the Republic of Korea during September 2010–August 2015. During three winter seasons, the density of infected *D. pulicaria* increased in all study sections, and they migrated from the central to the littoral area. Most of the infected individuals had dormant eggs in sexually reproducing mothers. However, when the proportion of non-infected individuals was higher than that of infected individuals, winter migration was not observed. Additional microcosm experiments showed that dormant eggs of *D. pulicaria* obtained from ice crystals in the littoral area had lower hatching and infection rates than those obtained from mothers moving from other zones. Therefore, the migration of *D. pulicaria* during winter is an active response to avoid intergenerational fungal infection.

## 1. Introduction

Biological interactions are important factors in determining the community composition and distribution of local organisms and the interrelationships among biological communities in the food web [1,2]. Various organisms coexist in preferred spaces by forming interrelationships, such as competition, predation, and infection, and maintain a balance among communities by obtaining survival opportunities and continuous population growth [3]. In general, a strong competitor or predator can acquire more resources [4], which leads to regional dominance and sustainability of a particular species or community [5]. However, the development of various evolutionary strategies has focused on lagging competitors and prey [6] because the few food acquisition failures of strong competitors or predators have less impact on their survival and population growth, unlike in lagging competitors or prey where food acquisition failure or consumption by predators is closely related to survival [7]. Phenotypic and behavioral plasticity has been demonstrated as a broad defensive mechanism by which various prey cope with threats [8]. If predators fail to acquire food in succession, which poses a serious threat to their survival and population growth, they respond to the defense strategies of the prey, but most predators that can acquire alternative food sources do not respond to them one by one [9]. Theoretically, a susceptible defense mechanism of prey may actively evolve when it is spatially or temporally challenged to survive from a predator and when a reliable clue to the future risk of these threats is understandable [10]. The defensive response of prey is not only a means that can be distributed in preferred areas, even in the presence of predators, but also a factor that increases regional biodiversity by coexistence with predators.

In freshwater ecosystems, cladocerans are a representative prey community utilized as a major food source by various predators, such as fishes and invertebrates [11,12]. The Cladocera community, with continuous movement and relatively low swimming speed, can be easily searched by predators. Therefore, this community has evolved various defense strategies to survive and maintain a certain density. The diel vertical or horizontal migration patterns of cladocerans are observed in various lakes, reservoirs, and wetlands as predator avoidance responses suitable for the habitat characteristics in each environment [13,14,15]. Visual predator fish cannot perform foraging activities at the bottom layer of deep lakes or reservoirs where light intensity is low or where the littoral area is complexly structured by aquatic macrophytes; therefore, the cladocerans residing at these areas can be protected from predatory threats [16]. In addition to these behavioral responses, morphological reactions, such as changes in the head or spin length of Cladocera species, are typical defense mechanisms that are observed in the presence of fish [17]; however, such changes are not very effective in avoiding predators. Furthermore, cladocerans protect their community composition and density from predators by changing their reproductive mode. Under good conditions, the Cladocera community continues to grow through asexual reproduction, but during challenges, such as shortage of food, low water temperatures, and the presence of predators, they switch to a sexual reproduction mode [18]. Dormant eggs produced by sexual reproduction have high physical and chemical resistance; therefore, they can survive poor environmental conditions. The flexible plasticity of cladocerans is a decisive obstacle for survival in the presence of various predators.

Previous studies have suggested various interactions, such as selective defensive responses of cladocerans to predators [19,20,21]. However, reports on other types of interactions, such as infections caused by parasites (e.g., Amoeba, Nematoda, and Cestoda), bacteria (e.g., *Pasteuria ramose* and *Spirobacillus cienkowski*), and fungi (e.g., *Metschnikowia bicuspidata*) are relatively scanty. This lack of information is surprising, considering that various biological communities, including cladocerans, are frequently affected by parasites in the natural ecosystem. Previous studies on infection in cladocerans have primarily focused on their survival and offspring production in laboratory settings [22,23,24], and very few interactions, such as their defense strategies against infection, have been studied. Empirical studies have tried to associate infection with cladocerans by considering the effects of single factors [25,26], such as food quality [27,28], nutrient availability [29], and water temperature [30], on their survival and mortality. For example, elevated water temperatures increase the abundance of certain food resources, such as cyanobacteria, and reduce the food quality of cladocerans [31]. Cyanobacteria lack steroids and polyunsaturated fatty acids, which are essential for cladocerans [32]. Therefore, domination of a specific food source may deteriorate host defense owing to stress caused by cyanotoxins, as well as a decrease in nutrient intake [33]. In addition, high temperatures can promote the absorption of fungal spores by increasing the filtration rate of cladocerans with an increased metabolic rate [34]. The spread of infection lowers the species diversity and abundance of cladocerans, while altering the outcome of competition among genotypes. For example, three parasite groups, *Metschnikowia* sp., microsporidia, and oomycetes, increase the opacity of the host; as a result, infected individuals can be easily identified by fishes performing foraging activities using their vision [35]. In deep lakes or reservoirs, if the infected host moves to the bottom layer to avoid fish predation, the infection may spread among individuals at the bottom layer [36,37]. Furthermore, *D*. *galeata* × *longispina* hybrids face a higher rate of infection by parasites than *D*. *galeata* and are, therefore, prone to extinction [38].

Infection affects the host more directly than predation. The effects of predation are widespread in prey groups, without the targeting of individuals. However, infections spread from a narrow to a wide range of species, such as targeting of specific individuals and communities [39]. Thus, a continuous arms race [40] between parasites trying to infect their hosts and hosts trying to avoid infection determines the survival and population growth of these two groups. One aspect of the Red Queen hypothesis [41,42] is that individuals with parasitic resistance genes can selectively survive in host populations. Moritz et al. [43] reported that sexual *Heteronotia binoei* is more susceptible to infection by mites than parthenogenetic individuals. These findings suggest that cloning does not induce continuous gene changes and, therefore, the individuals produced by cloning are likely to be eliminated during co-evolution. However, it is difficult for a host to determine its evolutionary direction by one influencing factor, such as parasitism. This is because various interactions in addition to infection have a complex effect on the survival and population growth of hosts. By contrast, a parasite only needs a successful infection of its host to survive [44]. Therefore, a parasite constantly invests energy to infect its host efficiently. This aspect of ‘Essential’ and ‘Selection’ in the parasite–host relationship results in a difference in the cost of energy input, ushering in the ‘Evolutionary Arms Race [40]’ between the two groups. Therefore, cases of successful avoidance of infection or the presence of resistant genes are mainly reported in rare or closed ecosystems.

Owing to the negative effects of fungal infection on survival and reproduction, cladocerans may possess various defense mechanisms to avoid vector infection; however, they are largely unexplored. We hypothesized that the winter migration pattern of *Daphnia pulicaria* observed in a small reservoir (Anri Reservoir, Republic of Korea) is a defense strategy to avoid fungal infection. In general, horizontal migration is a behavioral strategy for some Cladocera species to avoid predation by fish in shallow reservoirs [45,46]; however, their movement to the littoral area under low water temperatures may be less related to interactions, such as predation or competition. In this reservoir, we set three objectives through long-term monitoring of *D. pulicaria*: (1) seasonal changes in the distribution of infected or non-infected *D. pulicaria*; (2) winter migration pattern of sexual reproduction in *D. pulicaria* with fungal infection; and (3) effect of freezing of dormant eggs on the transmission of fungal infections between generations. To address these objectives, we investigated environmental variables and the Cladocera community, including *D. pulicaria*, in the Anri Reservoir, through long-term monitoring (September 2010–August 2015) and demonstrated the results of this field survey through laboratory experiments. The laboratory experiments were designed to assess the effect of freezing of dormant eggs in *D. pulicaria*, which moved to the littoral area during winter, on hatching and intergenerational infection rates. The findings explain the evolutionary strategies behind the migration of *D. pulicaria* to survive fungal infections.

## 2. Materials and Methods

### 2.1. Study Area

**Figure 1:**
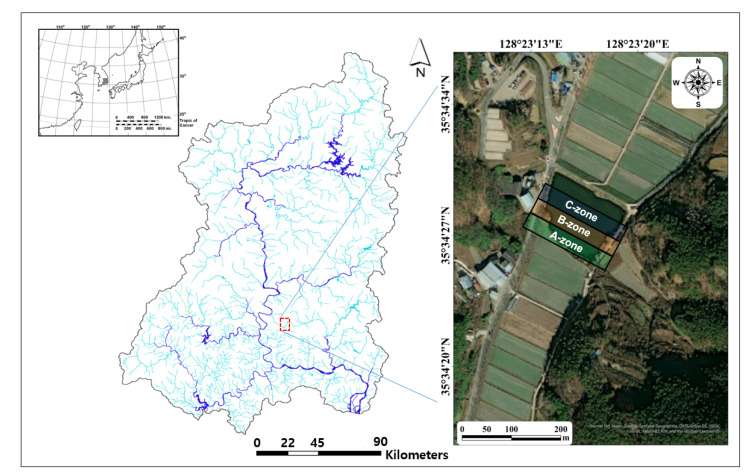
Map of the study site (Anri Reservoir) in the Nakdong River basin located in the southeastern part of the Republic of Korea. The small map in the upper left corner is the Korean Peninsula, and the sampling area (Nakdong River basin) is highlighted with a grey background. The red-dotted square in map of the Nakdong River basin is the study site and its surrounding area. The three regions (A-zone, B-zone, and C-zone) displayed in the satellite map indicate the sampling points.

The Nakdong River basin, where the study site (Anri Reservoir; Figure 1) is located in the southeastern part, is the longest and has the largest basin area among rivers and streams located in the Republic of Korea (length, 521.5 km; basin area: 23,817 km^2^). The upper part of the Nakdong River meets a forest area; therefore, the slope is steep and the water flow is rapid. The middle and lower reaches flow through the low hills and plains, maintaining a gentle water flow. The middle and lower reaches and the surrounding areas are covered by soil (i.e., alluvium) with poor drainage functions, and wetlands and ponds of various sizes are distributed owing to frequent flooding caused by concentrated summer rainfall. Many lentic ecosystems, such as the Upo Wetland and Jangcheok Lake, were distributed around the confluence of the Nakdong River and Namgang River, but most were destroyed during the 1970s because of policies aimed to expand agricultural and residential areas. The Anri Reservoir, a survey area, also has characteristics close to those of wetlands or ponds, where embankments are artificially trapped in areas with stagnant water owing to flooding.

The Anri Reservoir is located in the upper part of a small stream flowing into the Nakdong River and depends on the water source flowing from the forest area on the left and upper parts of the small stream. However, the water sources introduced from the upstream are intermittent and low in quantity, and little outflow downstream to maintain an appropriate water level is noticed; therefore, it is actually a closed ecosystem with little interaction with the outside. The area of the Anri Reservoir is relatively small compared to those of wetlands and reservoirs located in the Republic of Korea (14,191 m^2^) and is square-shaped. The littoral area is shallow with a depth of 0.1 to 0.5 m and is made of impermeable artificial materials, such as concrete and stone, making it difficult for shrubs, such as reeds (*Phragmites communis*) and willows (*Salix koreensis*) to settle, and its openness is very high. The structure of the Anri Reservoir gradually deepens from the littoral area to the center, with the deepest central part of approximately 2 to 3 m. The reservoir was reformed to secure water in adjacent agricultural lands, and human interference is relatively strong. We divided the study section into three areas based on the physical environmental characteristics (shape and depth) of the Anri Reservoir (Figure 1). The A-zone is the littoral area located in the southernmost part of the reservoir and has a depth of 0.1 to 0.5 m. The B-zone and C-zone are sequentially located toward the central direction of the reservoir, and the water depth is 0.5 to 1 m and 1.5 to 2.5 m, respectively. Each survey section is approximately 2.5 m wide, and the total survey width is 7.5 m from the littoral area to the center. We set nine sampling points, three for each of the study sections for long-term monitoring, and each sampling point was 1.5–2.0 m apart horizontally. Unlike the A-zone, the B-zone and C-zone were investigated by dividing them into surface (within 0.5 m from the surface) and bottom (within 0.5 m from the floor) layers because the water depth was relatively high.

### 2.2. Field Survey

We investigated environmental variables and the Cladocera community biweekly for five years from September 2010 to August 2015 at the nine sampling points. While the A-zone was investigated in littoral areas close to land, boats were used for the B-zone and C-zone, which are relatively deep. From the surface layers of the three zones, a 10 L water sample was collected using a 10-L column water sampler (length, 20 cm; width, 30 cm; height, 70 cm), and a Van Dorn water sampler was used for the bottom layers of the B-zone and C-zone. The water samples were immediately transported to the laboratory, 3 L was used to measure environmental variables, and the Cladocera community was collected from the remaining 7 L water samples. Four environmental variables, water temperature, dissolved oxygen (DO), chlorophyll a (chl. a), and turbidity were monitored. DO was measured using a DO meter (model 58; YSI Inc., Yellow Springs, OH, USA), and turbidity was analyzed using a turbidimeter (Model DRT 100 B, HF Scientific, Inc., Fort Meyers, FL, USA). The three-liter water sample was filtered through a mixed cellulose ester (MCE, Model No. A045A047A) membrane filter with a pore size of 0.45 µm (Advantech Co., Hyogo, Japan). The filtrates were used to determine the concentration of chl. a, based on the methodology of Wetzel and Likens [47]. In addition, the freezing time of the A-zone during winter was monitored.

The 7 L water sample for Cladocera collection was filtered through a plankton net (68-mm mesh), and the filtrate was preserved in sugar formalin (final concentration, 4% formaldehyde; [48]). The cladocerans were enumerated and identified at the species level using a microscope (ZEISS, Model Axioskop 40; 200× magnification), and the identification was based on the classification key published by Mizuno and Takahachi [49] and Thorp and Covich [50]. We checked the following points for *D. pulicaria* when the cladocerans were identified using a microscope: (i) individual characteristics (i.e., asexual, sexual, immature, and male) and (ii) fungal infections. Individual characteristics and fungal infections can be visually identified [51,52]. In particular, infected cladocerans are difficult to identify compared to non-infected individuals in the intestine, and black substances spread around the intestine and eyes are noticed. We evaluated *D. pulicaria* individuals in the cladoceran samples during the study period, based on the two factors described above.

We conducted additional investigations to identify the diel vertical migration patterns of *D. pulicaria* in each study section. The density of *D. pulicaria* during the day relied on the aforementioned field survey, and night surveys were conducted monthly during two periods (September 2010 to January 2011 and September 2011 to January 2012). *D. pulicaria* collected at night was also evaluated using the method described in the field survey during daytime.

To understand the influence of the distribution of *D. pulicaria* on fish predation, we investigated the seasonality of fish communities. Fishes were collected using cast (7 mm × 7 mm) and scoop (5 mm × 5 mm) nets along 200 m transects from the A-zone and C-zone twice in four seasons [winter (January), spring (May), summer (August), and autumn (October)] during the period from October 2010 to August 2015. The cast and scoop nets were used for 30 and 20 min, respectively. Fishes were not collected from the B-zone as it is adjacent to the A-zone and C-zone. Fish samples were identified to the species level according to Kim and Park [53] and the classification system of Nelson et al. [54]. Fish species that were difficult to identify in the field were fixed using a methanol–formaldehyde solution (3:1) and subsequently identified in the laboratory.

### 2.3. Microcosm Experiments

To determine the cause of horizontal migration of sexual *D. pulicaria* during winter, we conducted a hatching experiment using their dormant eggs. The dormant eggs were divided into two groups based on their origin: (1) eggs collected from the ice cover of the A-zone and (2) eggs collected from *Daphnia* mothers in the B-zone. Eggs in the first group were collected from ice crystals that had been frozen for at least 20 days during winter (January–February), and eggs in the second group were obtained from sexual *Daphnia* mothers in November. *Daphnia* mothers with dormant eggs collected in November were cultured in a laboratory environment and naturally induced to desorb eggs. The mother was housed in a plant growth chamber (Eyela FLI-301N, Tokyo, Japan) with 50 photon flux density (µmol·m^−2^·sec^−1^) and a 12:12 light–dark cycle. The eggs were kept dry at room temperature (20 °C) in a laboratory environment and used for experiments.

A microcosm experiment was designed to evaluate hatching and infection rates of the dormant eggs. We used 100-mL beakers for the entire experiment; algal food (i.e., chlorella) at a sufficient concentration [55] was provided to *D. pulicaria*, and a concentration of 4.5 × 10^5^ cells·mL^−1^ was considered sufficient for *D. pulicaria* survival and population growth (22.5 mg·CL^−1^). The culture medium used here was prepared using the collected water samples filtered twice using a 0.45 µm mesh. We confirmed the absence of algae or organic matter as food for *D. pulicaria* in the filtered water using a microscope (ZEISS, Model Axioskop 40, Oberkochen, Germany). To avoid pH changes owing to photosynthesis, we stored filtered water within a refrigerator (2 °C) and removed it just prior to experiments. Hatching experiments were conducted using a plant growth chamber (Eyela FLI-301N, Japan) with 50 photon flux density (µmol·m^−2^·sec^−1^) and a 12:12 light–dark cycle. Thirty beakers were selected per group, with 50 eggs in each beaker. Each day during the experiment, the eggs were transferred to fresh culture medium and food (algae) was provided to meet sufficient food condition. Experiments were conducted for approximately 10 days. The number of embryos per egg was counted using a microscope to estimate the hatching rate.

$$\mathrm{Hatching}\;\mathrm{rate}\;\left(\%\right)=\frac{\;\mathrm{number}\;\mathrm{of}\;\mathrm{hatching}\;\mathrm{individuals}}{\begin{array}{c} \;\mathrm{number}\;\mathrm{of}\;\mathrm{embryos}\;\mathrm{per}\;\mathrm{diapausing}\;\mathrm{egg} \end{array}}\times 100$$

To determine the infection rate, *D. pulicaria* hatched from the two experimental groups were maintained under culture conditions. The neonates were identified as infected or non-infected using a microscope after growth for five days.

$$\mathrm{Infection}\;\mathrm{rate}\;\left(\%\right)=\frac{\;\mathrm{number}\;\mathrm{of}\;\mathrm{infected}\;\mathrm{individuals}}{\begin{array}{c} \;\mathrm{number}\;\mathrm{of}\;\mathrm{hatching}\;\mathrm{individuals} \end{array}}\times 100$$

### 2.4. Data Analysis

Stepwise multiple regression analysis was performed to examine the relationship of *D. pulicaria* density with the four environmental variables. Analyzing the relationship between the abundance of the remaining Cladocera species except *D. pulicaria* and the environmental variables was difficult because an annual average density of <50 ind./L was observed. Furthermore, one-way analysis of variance (ANOVA) (α = 0.05) was used to determine the statistical differences in (i) the four environmental variables and the abundance of *D. pulicaria* between the surface and bottom layers and in the (ii) abundance of *D. pulicaria* during day and night.

For statistical analyses of the hatching experiment, we applied a one-way nested ANOVA (two-tailed, a = 0.05) to identify the hatching and infection rates. Although we prepared 20 replicates for each experimental group, pseudo-replication required careful consideration (i.e., data homogeneity between the replicates of each experimental group needed to be ensured) [56]. Therefore, we set different experimental groups as the primary factors and 20 replicates as nested subgroups for each treatment. Furthermore, we analyzed the normal distribution and variance homogeneity to verify the previous conditions for ANOVA [57,58].

Because the main focus of this study was to evaluate the delayed influence of the density of infected *D. pulicaria* in the A-zone on the increased density of infected *D. pulicaria* in the C-zone during three winters, we also used a time-series analysis method in ‘Cross-Correlation Analysis (CCA)’. Biweekly data in the A- and C-zone were compared, and correlation factors between the densities of infected *D. pulicaria* in the A- and C-zone were obtained.

All statistical analyses were conducted using SPSS ver. 20 (released 2011; IBM SPSS Statistics for Windows, Version 20.0. Armonk, NY, USA: IBM Corp.). Differences and relationships were considered significant at *p* < 0.05.

## 3. Results

### 3.1. Environmental Variables and D. pulicaria

**Figure 2:**
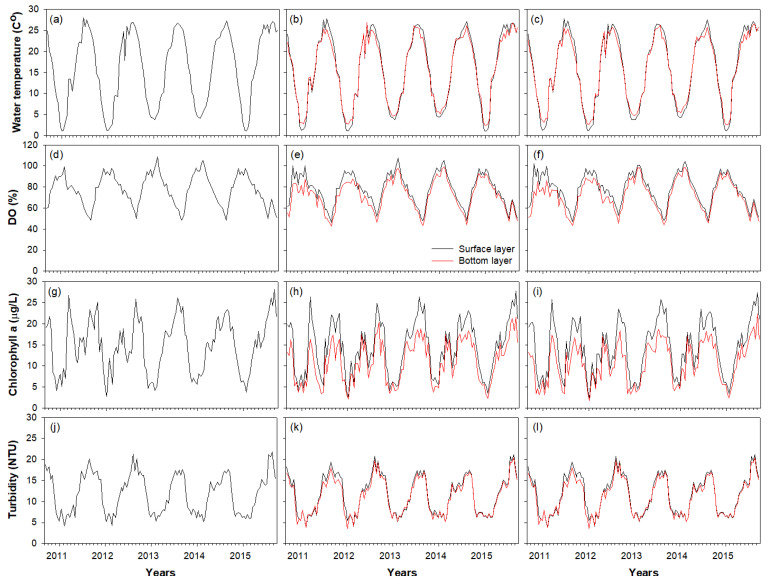
Time-series fluctuations (September 2010–August 2015) of environmental variables [water temperature, dissolved oxygen (DO), chlorophyll a, and turbidity]. Water temperature in the A-zone (**a**), B-zone (**b**), and C-zone (**c**); DO in the A-zone (**d**), B-zone (**e**), and C-zone (**f**); chlorophyll a content in the A-zone (**g**), B-zone (**h**), and C-zone (**i**); turbidity in the A-zone (**j**), B-zone (**k**), and C-zone (**l**). Blue bars indicate the period during which the A-zone was frozen.

The four environmental variables measured in each study section exhibited a typical seasonal pattern in the temperate climatic zone (Figure 2).

The water temperature, turbidity, and chl. a content were high in summer (June–August) and low in winter (December–February), whereas DO levels showed an opposite pattern. The water temperature reached 25–27 °C during summer, while it ranged between 1 and 5 °C during winter. The A-zone was frozen three times (January–February in 2012, 2013, and 2016) during winter, and surface water temperature at this time was 1–2 °C. However, the A-zone was not frozen during winter (January–February in 2014 and 2015) when relatively high water temperatures (3–5 °C) were maintained. DO levels ranged from 45 to 110%, and chl. a and turbidity values showed relatively small fluctuations at 3–27 ug/L and 5–21 NTU, respectively. These environmental variables differed between high and low temperature situations; however, a similar seasonal pattern was observed during the five–year study period. The seasonal differences in environmental variables in the three zones and water layers (surface and bottom) were not significant (one-way ANOVA, *p* > 0.05). However, in summer and autumn, DO and chl. a levels were higher in the surface layer than in the bottom layer (one-way ANOVA, *p* < 0.05).

Five Cladocera species were identified during the study. *D. pulicaria* was the most dominant species at the study sites (relative richness, 84%), followed by *Chydorus sphaericus* (10%). The relative richness of the remaining cladoceran species was <10% (*Simocephalus vetulus*, 3%; *Pleuroxus aduncus*, 1.6%; *Simocephalus expinosus*, 1.4%).

**Figure 3:**
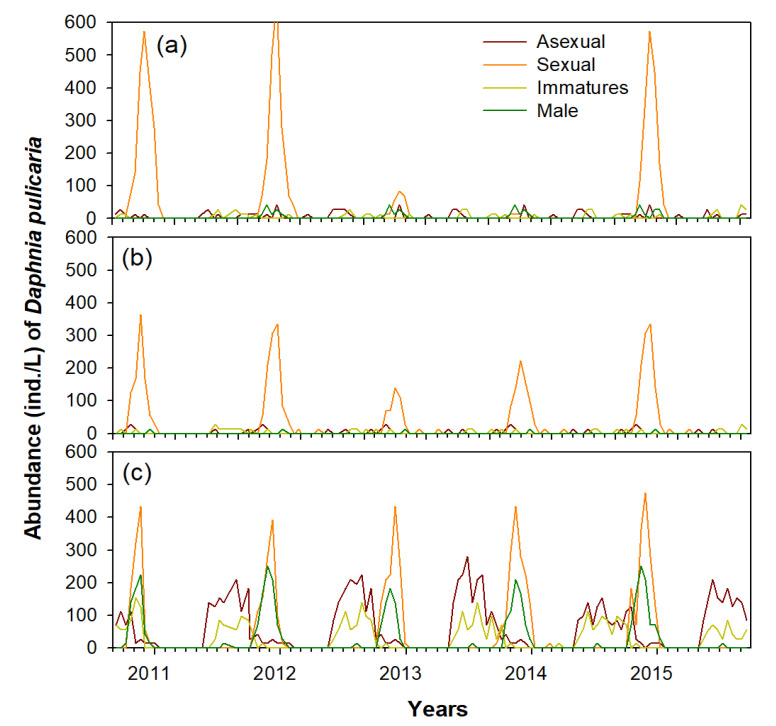
Interannual changes (September 2010–August 2015) in the abundance of *D. pulicaria* in accordance with individual characteristics (asexual, sexual, immatures, and male). (**a**) A-zone, (**b**) B-zone, and (**c**) C-zone.

The seasonal distribution pattern of the dominant species, *D. pulicaria*, differed in each study section (Figure 3). In the bottom layer of the C-zone, *D. pulicaria* was observed from June to December, while in the A-zone and B-zone, it was mainly distributed in winter only (December–February).

*D. pulicaria* was abundant in winter in the A-zone, and a relatively more abundance was noticed in three of the five winters (December 2010, 2011, and February 2014). In contrast, the density of *D. pulicaria* in the bottom layers of the B-zone and C-zone was similar during all winters. The surface layer had a very low abundance of *D. pulicaria*. From the density pattern observed during October–February, movement of *D. pulicaria* from the C-zone to the B-zone and finally to the A-zone was evident (Figure 3). The density of *D. pulicaria* gradually decreased after peaking in November in the C-zone and increased in the B-zone, and finally increased in the A-zone during January–February.

**Figure 4:**
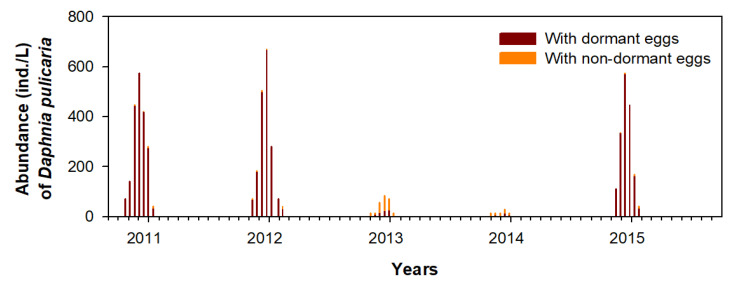
Abundance of *Daphnia pulicaria* with dormant and non-dormant eggs in the A-zone.

**Figure 5:**
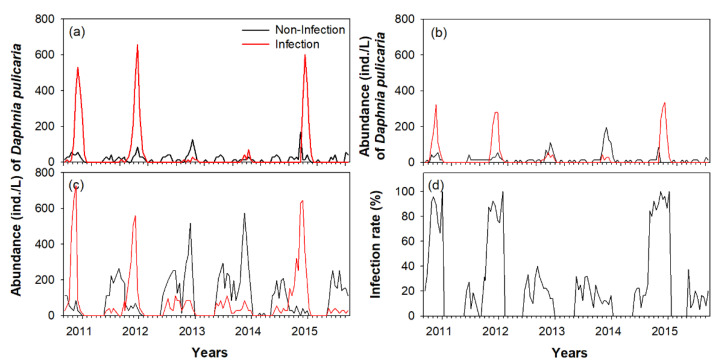
Interannual change (September 2010–August 2015) in non-infection and infection abundance of *Daphnia pulicaria* in each study section (A-zone, B-zone, and C-zone) of the Anri Reservoir. (**a**) A-zone, (**b**) B-zone, (**c**) C-zone, and (**d**) relative infection rate in the C-zone.

During winter, *D. pulicaria* distributed in each study section was mostly sexually reproducing females (Figure 4), mainly observed in three (2010, 2011, and 2015) of the five winters in the A-zone, while in the bottom layers of the B-zone and C-zone, it presented similar densities during all winter periods. During winter, most of the females concentrated in the A-zone were sexually reproducing mothers with dormant eggs (Figure 5). The immature individuals and males were relatively abundant in the C-zone during June–October; however, their abundance was very low in the A-zone and B-zone. In the C-zone, between June–October, *D. pulicaria* was dominated by asexually reproducing females, and the number of sexually reproducing females was very small. The density of the asexually reproducing females sharply decreased after an increase in the density of males in mid-October, and from November, sexually reproducing females were dominant. This seasonal density pattern of *D. pulicaria* did not significantly differ between night and day (one-way ANOVA, *p* > 0.05; Table 1).

### 3.2. Distribution Pattern of Infected Individuals

**Figure 6:**
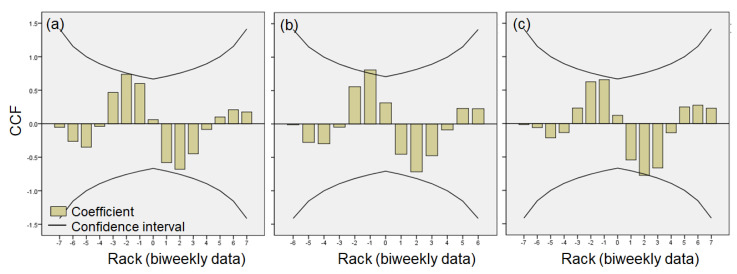
Cross correlation factor (CCF) in infection abundance of *Daphnia pulicaria* in two study sections (A-zone and C-zone) of the Anri Reservoir. (**a**) A-zone, (**b**) B-zone, and (**c**) C-zone.

In each study section, a high density of infected *D. pulicaria* was observed in winter (Figure 5). In the A-zone, where high density was observed during three winters (2012, 2013, and 2016), *D. pulicaria* females were mostly infected; during this period, the infected individuals were also abundant in the B-zone and C-zone. The difference in density between infected and non-infected individuals in the A-zone during the three winters was statistically significant (one-way ANOVA, *p* < 0.05). During the two winters in 2013 and 2014, *D. pulicaria* in the B-zone and C-zone were mostly non-infected, and in the A-zone, the densities of infected and non-infected individuals were low. In the C-zone, *D. pulicaria* population, which was abundant in all seasons except for winter, was dominated by non-infected individuals. During the three winters in 2012, 2013, and 2016, the infection rate of *D. pulicaria* was more than 90%, whereas it was less than 30% in the winters of 2014 and 2015 in the C-zone (Figure 6c).

The application of cross correlation to infected density in the A-zone over the infected density of *D. pulicaria* in the C-zone revealed a significant time-series relationship (Figure 6). The high density of infected *D. pulicaria* in the C-zone during the three winters generally had a significant negative impact on the changes in infected *D. pulicaria* density in the A-zone, and the period of delay was approximately one or two weeks. This relationship was found in all three winters.

### 3.3. Seasonal Distribution of Fish Community

Three fish species, *Micropterus salmoides*, *Lepomis macrochirus*, and *Pseudorasbora parva*, were collected from the Anri Reservoir, and different temporal and spatial distribution characteristics were observed (Table 2). These fishes were mainly collected during spring–autumn and were absent or very scarce in winter. Only a small number of *L. macrochirus* was found in winter. During the study period, *M. salmoides* was collected in the C-zone, and *L. macrochirus* and *P. parva* were mainly found in the A-zone.

### 3.4. Hatching and Infection Rates in Dormant Eggs

**Figure 7:**
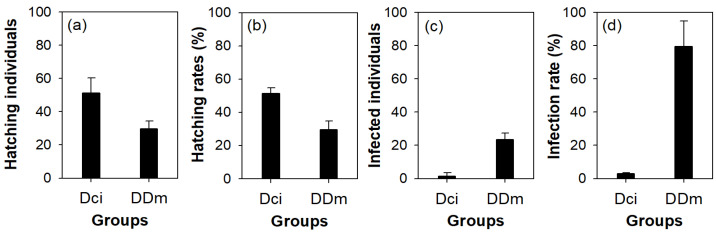
The hatching and infection rates in dormant eggs of *D. pulicaria* in the two groups (Dci and DDm). Dci, dormant eggs collected from the ice cover; DDm, dormant eggs collected from *Daphnia* mother. (**a**) Hatching individuals, (**b**) hatching rates, (**c**) infected individuals, and (**d**) infection rates of dormant eggs of *D. pulicaria*.

The hatching and infection rates in dormant eggs of *D. pulicaria* significantly differed between the two groups [resting eggs collected from the ice cover in the A-zone (Rci) and from mother (RDm)] (Figure 7 and Table 3). Hatched populations and hatching rates of dormant eggs were higher in Rci than in RDm, whereas infected populations and infection rates among hatched populations were lower in Rci than in RDm. The differences in incubation and infection rates of dormant eggs between the two groups were statistically significant.

## 4. Discussion

### 4.1. Spatial Distribution of Daphnia pulicaria

In the Anri Reservoir, the seasonal distribution of *D. pulicaria* differed according to the study sections (A-zone, B-zone, and C-zone) and water layers (surface and bottom layer). In the bottom layer of the C-zone, *D. pulicaria* was distributed from June to January every year. In contrast, in the bottom layers of the A-zone and B-zone, *D. pulicaria* was found in winter only (December to February) and was rarely observed in other seasons (spring, summer, and autumn). The surface layers of the B-zone and C-zone had low densities of *D. pulicaria*. Although these different spatial distribution patterns can be interpreted from various aspects, including biological factors, such as competition, predation, and food source, and non-biological factors, such as physicochemical variables and nutrients [59,60], the effect of predators is assumed to be the most influencing factor based on the environmental characteristics of the Anri Reservoir. The three fish species found in the study site frequently use cladocerans, including *D. pulicaria*, as food sources [61,62]. Therefore, in freshwater ecosystems, the spatiotemporal distribution of the Cladocera community is closely related to the foraging activities of predators, such as fish [63]. In particular, species belonging to the genus *Daphnia* (e.g., *D. galeata*, *D. obtusa*, and *D. pulex*) are utilized as a major food source for fish [64,65]; therefore, the density of cladoceran species, including *D. pulicaria*, is low during spring–autumn, when predators are active [66]. Therefore, some cladoceran species avoid predators by distributing themselves in spaces where predators are reluctant to go (e.g., areas with low DO or phytoplankton) or face difficulties in search for food. The bottom layers of lakes and reservoirs [67,68] and spaces covered by aquatic macrophytes [69,70,71] have been suggested as representative refuge spaces for cladocerans to avoid predators. Fish that search for food using vision restrict themselves from foraging activities in spaces complexly created by the leaves and stems of aquatic macrophytes or deep-water levels with low light penetration.

In this study, *D. pulicaria* was more abundant in the bottom than in the surface layers to avoid predators. However, for the bottom layer of a lake or reservoir to become a refuge for cladocerans, light must be introduced and DO levels should be low [72,73]. Fish distribution might be limited when DO is less than 20% in the bottom layer [74]. The maximum water depth of the Anri Reservoir is 2.5 m, which is relatively low, and DO levels are not significantly different between the surface and bottom layers, raising a concern regarding the efficiency of bottom layers as refuge for *D. pulicaria*. However, the high turbidity in the bottom layer of the Anri Reservoir may render it suitable as a refuge.

Kim and Choi [68] suggested that high turbidity at the bottom layer of an estuary of the Nakdong River, which has a relatively low water depth, induced diel vertical migration of *D. obtusa*. Inorganic or organic materials transported from the upper and middle streams of the Nakdong River are accumulated in this area, and turbidity of the bottom layer is high owing to the high residence time of those materials. A bottom layer with high turbidity may disturb foraging activities of predators, such as fish [75,76]. The high turbidity of the bottom layer of the Anri Reservoir might be caused by the promotion of inflow of turbid materials from upstream and surrounding agricultural lands during concentrated summer rainfall. The turbidity of the bottom and surface layers of the Anri Reservoir is similar; however, foraging activity of fish is easy at the surface layer owing to a comparatively high light intensity.

We did not observe a vertical migration pattern of *D. pulicaria*. Little difference in the densities of *D. pulicaria* was observed in the surface and bottom layers of the C-zone between day and night. Phytoplankton, which is used as a food source for *D. pulicaria*, is probably abundant in both layers; therefore, movement to the surface layer to acquire food at night may not be necessary. Although the chl. a concentration in the bottom layer of the C-zone was lower than that in the surface, it remained high enough and enabled *D. pulicaria* to lower the energy cost of moving to the surface layer at night.

### 4.2. Winter Migration of Infected Hosts

*D. pulicaria* gradually migrated from the C-zone to the A-zone in early winter (November–December). Sexually reproducing *D. pulicaria* began to increase in the C-zone in November, decreased in December–January, and increased in density in the B-zone or A-zone (January–February). The very low density in the B-zone and C-zone during the peak period (January–February) indicates that *D. pulicaria* moved to the A-zone. *D. pulicaria* was more abundant in the A-zone than in the B-zone and C-zone during peak periods as individuals scattered in the bottom layer of other regions were concentrated in the littoral area (i.e., A-zone). This migration pattern was observed three times (January–February in 2012, 2013, and 2016). *Daphnia* species migrate when predators, such as fish and invertebrates, exist, and most of them complete a cycle of migration patterns within 24 h. However, migration over a relatively long period of time, as in this study, has not been observed in previous studies, which shows the possibility of exploring a new behavioral strategy of *Daphnia* species by the present study.

The winter migrations of *D. pulicaria* were caused by individuals infected with fungal parasites (*Metschnikowia* sp.). The CCA analysis showed that the density of infected *D. pulicaria* increased in the C-zone during the three winters in which winter migration was closely related to the density of infected *D. pulicaria* in the A-zone. The proportion of infected individuals in the three winters in which *D. pulicaria* migrated was more than 80%. In contrast, relatively few infected individuals were noticed during the two winter periods without migration of *D. pulicaria*. Therefore, infection was the main cause of winter migration of *D. pulicaria*. Although clearly demonstrating the migration pattern of *D. pulicaria* as a factor dependent on infection rates was not possible, the bottom layer of the C-zone, where the threat of infection disappeared in winter, continuously provides *D. pulicaria* with positive environmental conditions (e.g., predator avoidance, mating, or food acquisition). However, during infection spread, continuous concentration of *D. pulicaria* in the C-zone can lead to generational transmission of infections; therefore, the migration of infected individuals to other regions may be a smart survival strategy for the sustainability of individuals.

However, even in vertical migration in winter, when the density of *D. pulicaria* was low owing to their migration to the A-zone, males and sexually reproducing females were still frequently observed in the C-zone, therefore making it difficult to determine whether fungal infection affected reproductive mode change of *D. pulicaria*. The reproductive mode change of cladocerans (from asexual to sexual) is related to changes in physicochemical factors [77], such as water temperature and nutritional salt, as well as biological factors [78], such as predator survival and food reduction. Fungal infection also majorly contributes negatively to the maintenance of density and population growth of cladocerans, and although it might cause reproductive mode changes, no conclusive evidence has been found in this study. Given that the time where fungal infection spread in the Anri Reservoir is autumn or winter, the gradually lower water temperature during this period also acts as a strong disturbing factor for *D. pulicaria*. Therefore, the effect of fungal infection may overlap or be hidden by the effect of low water temperature. We observed the occurrence of males and mothers with sexual reproductive mode in autumn and winter every year, regardless of the spread of fungal infections in the C-zone.

*D. pulicaria* that migrated to the A-zone were mostly sexually reproductive mothers with dormant eggs. This means that they already mated before moving to the A-zone. During summer and autumn (June–November), the asexually reproductive mothers distributed in the C-zone produced male individuals before winter, leading to the generation of dormant eggs in sexually reproductive mothers. The fact that *D. pulicaria* males were observed mainly in the C-zone during winter means that mating in the A-zone and B-zone was unlikely to occur in addition to that in the C-zone. Mating attempts in the C-zone before moving to the A-zone during the spread of infection are considered strategies to minimize potential threat factors, such as the risk of predation or lack of food sources in the A-zone. Although the infection spreads, mating attempts in areas secure the species from predators (in the bottom layer of the C-zone since June) and can contribute to high viability and stable population growth. Furthermore, even if a mother with dormant eggs after mating dies for any reason while moving to the A-zone, survival of the population is feasible owing to the potentially resistant dormant eggs that can survive later. The A-zone was more disturbed (e.g., predation, competition, and freezing) than the B-zone or C-zone, and the survival of *D. pulicaria* mothers was uncertain; therefore, the dormant egg strategy is suitable to avoid fungal infections.

However, during winter migration, *D. pulicaria* cannot completely avoid fungal infection because individuals or dormant eggs produced by the infected mothers can inherit fungal infection. Vertical infection spread among infected individuals can cause a rapid decline in populations and may lead to extinction [79]. Although winter migration patterns may be efficient in preventing horizontal spread of infection away from the bottom layer of the C-zone, blocking intergenerational infection of *D. pulicaria* in winter is difficult. Therefore, another defense strategy is needed to prevent infection and secure the health of offspring of infected mothers.

### 4.3. Evolutionary Strategies to Prevent Horizontal Fungal Infections

The results of microcosm experiments can appropriately explain the reasons for winter migration patterns of infected mothers with dormant eggs in the Anri Reservoir. Although winter migration of these infected mothers is interpreted as a defense strategy to prevent the spread of fungal infections, concentration in the littoral area rather than local random spread is believed to be an advantage for *D. pulicaria* against fungal infections in the A-zone. We focused on mothers with dormant eggs, and dormant eggs that reached the A-zone and remained frozen during winter. In this study, the A-zone remained frozen for approximately 20–30 days during the three winters (January–February in 2012, 2013, and 2016), when winter migration of *D. pulicaria* was observed. The dormant eggs collected from ice crystals had a lower rate of vertical fungal infection than unfrozen dormant eggs (dormant eggs obtained from *D. pulicaria* mothers were distributed in the B-zone and C-zone in early winter). Therefore, the migration of *D. pulicaria* to the littoral area is an effective strategy to lower the infection rate of the next generation. Previous studies have also noticed this behavioral strategy of *D. pulicaria*. Behavior responses, such as vertical migration pattern [13,80] between the surface and deep-water layers in deep lakes or moving waterfront and center (horizontal migration pattern) [39]) in shallow wetlands, are strategies to avoid predators. For *Daphnia*, which has an efficient defensive behavior strategy called migration patterns, if fungal infections can be avoided in a similar way, it can reduce the cost of developing new defensive strategies by reducing energy consumption. Because fungal spores are vulnerable to freezing, dormant eggs are maintained in ice crystals for a long time; therefore, the extinction of fungal spores present in infected individuals can be expected [81]. Freezing in lakes or reservoirs occurs mainly in the surface layer or littoral area with shallow water depth; therefore, migration to the littoral area is easy unless *D. pulicaria* is distributed in the surface layer of the C-zone for a long time. The freezing of dormant eggs is suitable for avoiding intergenerational infections, given that the infection rate of hatched individuals in dormant eggs obtained from ice crystals in the A-zone was less than 1%. This experiment was conducted in a limited space in a beaker, and frequent contact between individuals may have further increased the infectivity of hatched individuals in dormant eggs obtained from ice crystals.

Infection, which is similar to interactions, such as predation or competition, can induce defensive strategies in prey for survival and resistance. Like predation and competition, infection is also a frequent biological interaction in the Cladocera community and has a decisive impact on population growth; therefore, appropriate defense strategies are essential to ensure population sustainability. However, the defense strategies observed in this study may be rare because the Cladocera community, including *D. pulicaria*, has been distributed in each freshwater ecosystem for a long time and has already formed acceptable interrelationships with various biological communities. Because freshwater ecosystems are fragmented and less connected than other ecosystems, the types of defense strategies and responses vary depending on how they interact with different types of biological communities within closed systems. The winter migration of *D. pulicaria* may be a defensive strategy limited to closed reservoirs in South Korea.

## 5. Conclusions

We suggest that the winter migration of *D. pulicaria*, observed in a small reservoir (Anri Reservoir) located in South Korea, is a defense strategy to avoid fungal infections. We further confirm the benefits of frozen dormant eggs in minimizing fungal infection, which was interpreted as an evolutionary defense strategy of *D. pulicaria* to avoid horizontal infection spread as well as vertical infection between generations through the freezing of dormant eggs and their spread. This infection avoidance is a flexible survival strategy for cladocerans for stable population growth. Strategies other than behavioral responses may be exhibited by Cladocera communities depending on the type of threats they encounter. Therefore, cladocerans are widely distributed worldwide in areas where they present various defensive responses against predation, competition, and infection.

## Figures and Tables

**Table 1:** Abundance of *D. pulicaria* during the day and night.

Year	Month	A-Zone		B-Zone (Surface)		B-Zone (Bottom)		C-Zone (Surface)		C-Zone (Bottom)	
		Day	Night	Day	Night	Day	Night	Day	Night	Day	Night
2010	September	42 ± 6.3	40 ± 5.2	-	-	14 ± 4.2	11 ± 1.5	-	-	168 ± 14	164 ± 23
	October	70 ± 5.2	73 ± 8.3	-	-	154 ± 13	149 ± 15	14 ± 1.6	12 ± 1.1	518 ± 25	520 ± 21
	November	448 ± 20	451 ± 18	-	-	364 ± 24	355 ± 21	28 ± 6.7	31 ± 7.2	812 ± 30	809 ± 26
	December	420 ± 23	426 ± 21	-	-	70 ± 9.4	58 ± 10	14 ± 3.6	16 ± 2.4	42 ± 5.1	37 ± 20
2011	January	42 ± 6.3	48 ± 6.3	-	-	-	-	-	-	-	-
	September	28 ± 3.2	25 ± 8.2	14 ± 2.3	15 ± 2.5	14 ± 2.1	16 ± 4.2	14 ± 2.2	14 ± 2.3	210 ± 8	205 ± 16
	October	14 ± 1.6	10 ± 2.1	-	-	14 ± 1.8	15 ± 3.6	-		98 ± 6.3	93 ± 11
	November	84 ± 9.2	80 ± 6.3	-	-	84 ± 6.5	79 ± 2.7	14 ± 2.7	14 ± 2.7	350 ± 12	346 ± 15
	December	518 ± 16	511 ± 25	-	-	308 ± 16	286 ± 18	28 ± 2.6	28 ± 2.4	630 ± 19	621 ± 31
2012	January	294 ± 13	284 ± 21	-	-	98 ± 11	93 ± 8.2	14 ± 3.1	14 ± 2.6	56 ± 6.7	52 ± 3.4

**Table 2:** Abundance of *Daphnia pulicaria* during day and night in each study section (A-zone, B-zone, and C-zone) of Anri Reservoir. *M. salmoides*, *Micropterus salmoides*; *L. macrochirus*, *Lepomis macrochirus*; and *P. parva*, *Pseudorasbora parva*.

Years	Fish Species	Winter (January)		Spring (May)		Summer (August)		Autumn (October)	
		A	C	A	C	A	C	A	C
2010	*M. salmoides*				1		2		2
	*L. macrochirus*	1		2		2		2	
	*P. parva*			1				1	
2011	*M. salmoides*				2		1		2
	*L. macrochirus*			2		1		1	
	*P. parva*								
2012	*M. salmoides*				1		2		1
	*L. macrochirus*	1		1		1		1	
	*P. parva*								
2013	*M. salmoides*						1		1
	*L. macrochirus*	1		3		1		2	
	*P. parva*							1	
2014	*M. salmoides*				1		2		3
	*L. macrochirus*			1		3		3	1
	*P. parva*			1				1	
2015	*M. salmoides*		1	1	2	1	1		2
	*L. macrochirus*			2		3		2	
	*P. parva*			1				1	

**Table 3:** Two-way nested analysis of variance (ANOVA) results for the effects of main groups (i.e., two groups; (i) dormant eggs collected from the ice cover (Dci), and (ii) dormant eggs collected from *Daphnia* mother (DDm)) and subgroups (i.e., 20 replicates) on hatching and infection rates in dormant eggs of *Daphnia pulicaria*.

Factors	Variance	d.f.	F	*p*
Hatching rates (%)	Groups	2	24.516	<0.001
	Beakers	28	0.488	>0.05
Infection rates (%)	Groups	2	87.219	<0.001
	Beakers	28	0.115	>0.05

## Data Availability

The data presented in this study are available upon request from the corresponding author. The data are not publicly available because of restrictions on the right to privacy.
